# Distribution Characteristics and Risk Assessment of Heavy Metals in Soil and Street Dust with Different Land Uses, a Case in Changsha, China

**DOI:** 10.3390/ijerph182010733

**Published:** 2021-10-13

**Authors:** Yalei He, Yan Zhang, Chi Peng, Xinxing Wan, Zhaohui Guo, Xiyuan Xiao

**Affiliations:** 1 School of Metallurgy and Environment, Central South University, Changsha 410083, China; linxcsu@foxmail.com (Y.H.); 213501065@csu.edu.cn (Y.Z.); zhguo@csu.edu.cn (Z.G.); xiaoxy@csu.edu.cn (X.X.); 2 Third Xiangya Hospital, Central South University, Changsha 410083, China; wanxinxing@csu.edu.cn

**Keywords:** urbanization, urban soil, health risk assessment, urban functional areas, monitoring analysis

## Abstract

Rapid urbanization and industrialization have led to the accumulation of heavy metals in urban areas. The distribution and health risk of heavy metals in soil and street dust were studied by collecting the samples in pairs from different land uses in Changsha, China. The results showed that the average contents of the heavy metals Pb, Cd, Cu, Zn, Cr and Ni in the soil were 45.3, 0.69, 46.3, 220.4, 128.7 and 32.9 mg·kg^−1^, and the corresponding heavy metal contents in the street dust were 130.1, 3.9, 130.8, 667.2, 223.2, 50.5 mg·kg^−1^, respectively. The soils in the parks and roadsides have higher heavy metal contents than those in the residential and agricultural areas. The street dust collected from parks, roadsides and residential areas contained higher heavy metal contents than agricultural areas. Significant correlations were found between heavy metals, suggesting similar sources. However, most of the heavy metals in the soil were uncorrelated with those in the street dust. The contents of heavy metals in soil are the results of long-term pollution. Street dust is easily affected by natural or human disturbances, reflecting pollution emissions in a short period. The health risks posed by heavy metals in the soil are acceptable, but the street dust may threaten children’s health, especially in residential areas. Pb, Cr and Cd are the main risk contributors. Reducing the emissions from industrial plants and traffic may reduce the risk of exposure to heavy metals in the street dust.

## 1. Introduction

Heavy metals are common persistent toxic pollutants in the environment [1], released from both anthropogenic sources and natural sources, such as the burning of fossil fuels, wear of vehicle tires and brake pads, erosion of paint and coatings, metal mining and smelting activities, and soil parent material [2,3,4]. The rapid urbanization, industrial production and the number of motor vehicles increasing exponentially, causes a large amount of heavy metals to be released into the environment [5]. Street dust, one of the main carriers of heavy metals in the urban environment, originates from building and road pavement erosion, industrial smoke, and traffic dust [6,7,8]. Soil has a high ability to adsorb heavy metals, thereby accumulating heavy metals continuously [5,9]. Heavy metals in soil and street dust will threaten the health of populations through accidental oral intake, dermal contact, and inhalation [10,11,12]. Knowledge of the characteristics, distribution and risk assessment of heavy metals in urban soil and street dust has thus become important.

The contents of heavy metals in urban soil are mainly affected by the type and intensity of surrounding pollution sources. Traffic emissions will significantly increase the content of heavy metals in the soil within 10 m from the road, but the impact beyond this distance becomes small [13]. The intensity of the regional baseline deposition of heavy metals is highly correlated with traffic and population density [14]. Hence, heavy metal contents in urban soils are higher than that in suburban and rural soils [15]. Land use type is a dominant factor affecting the contents of heavy metals in urban soils. The content of heavy metals in the soil in urban industrial and transportation areas is often higher than that in residential and rural areas [16,17]. The heavy metal contents in the soil change slowly [18], reflecting long-term pollution from surrounding sources.

Compared with soil, street dust tends to have relatively higher contents of heavy metals, posing a higher potential health risk to humans [19]. The primary uses of roads considerably affect the contents of heavy metals in street dust [20]. For example, the contents of heavy metals in street dust collected from roadsides of industrial transportation were higher than that from the other areas [21,22]. The contents of heavy metals in street dust are affected by natural environmental changes (such as rainfall and wind) and human activities (such as cleaning, washing, etc.) [23]. These disturbances significantly reduce the heavy metal content in street dust [6]. Street dust will enter the surrounding soil through atmospheric deposition or surface runoff, and can be regarded as a major source of heavy metals in urban soil [6,7,8]. The characteristics of heavy metals in street dust reflect short-term emissions, making them helpful to understand current urban pollution sources. 

The land use type affects the intensity of emission sources and human activities, and it becomes an important factor that indirectly affects the content of heavy metals in urban soil [20,24]. However, few studies have investigated the impact of land use type on heavy metal contents in both soil and street dust. Previous research on heavy metals in urban soil and street dust mainly concentrate on one phenomenon, but studies on the similarities and differences of the heavy metals between them are lacking. In this study, soil and street dust samples were collected in pairs from different land use areas in Changsha City to (1) study the characteristics and health risks of heavy metals in urban soil and street dust with different land uses; and to (2) compare the distributions of heavy metals in urban soil and street dust to provide scientific support for urban pollution prevention.

## 2. Materials and Methods

### 2.1. Study Area and Sampling

The city of Changsha, located in central China, has been facing rapid urbanization in the past decades, with both population and GDP increasing exponentially. The population and GDP of Changsha in 1978 were 4.58 million and 4.45 billion yuan, respectively, while in 2018 they were 7.29 million and 1214.3 billion yuan, respectively [25]. In 1994, the number of motor vehicles in the city was 123,618, and in 2020 it was 2,677,132. The primary industries in Changsha are metal smelting, equipment manufacturing and biotechnology. In 2018, a total of 219 companies in the metal smelting and processing industry, 527 companies in petroleum and chemical products manufacturing, 93 companies in pharmaceutical manufacturing and 326 companies in metal products and automotive manufacturing were recorded in Changsha [25]. The rapid growth of industrial production and the number of motor vehicles in Changsha would inevitably release a large amount of heavy metals into the surrounding environment. 

**Figure 1:**
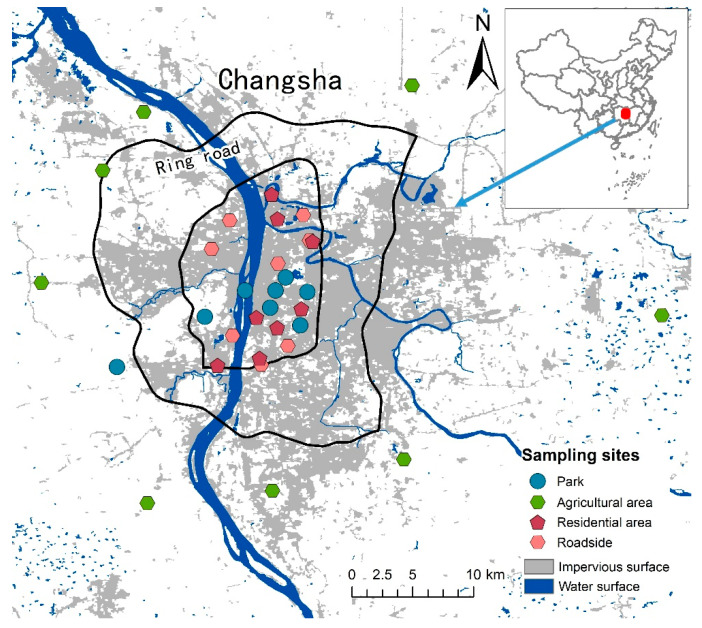
Sampling sites of urban soil and street dust in Changsha.

This study takes Changsha City as a case and selects four typical land uses as sampling sites, including the park, a residential area, roadside and a suburban agricultural area. Eight sampling sites were selected for each land use, and a total of 32 soil samples and 32 street dust samples were collected (Figure 1). Historical remote sensing images were used to ensure that the selected sampling sites have not undergone changes in land use for more than ten years. The samples were collected in March 2018, avoiding the rainy and strong wind seasons. Each surface soil (0–10 cm) sample was collected as a mixture of 5 subsamples taken within 100 m^2^ at the sampling sites. The street dust samples were collected using a vacuum cleaner with a high efficiency particulate air filter on the cement surface close to the soil sampling sites.

### 2.2. Chemical Analysis

The soil and dust samples were digested using the HNO3-HClO4-HF method. Samples were eeighed 0.20 g of soil or dust sample into a PTFE crucible, added 10 mL of nitric acid, 8 mL of hydrofluoric acid, and digested at 135 °C for 2 h, then added 2 mL of perchloric acid and digested at 160 °C for 50 min. Depending on the digestion progress, 2 mL of nitric acid, 2 mL of hydrofluoric acid, and 1 mL of perchloric acid was added to the crucible, and the above process was repeated until the organic matter disappeared. The temperature was raised to 175 °C until the remaining digestion solution was approximately 1 mL, then it was filtered and diluted to 25 mL with ultrapure water, waiting for quantitative determination. Blank samples and parallel samples were carried out for each batch of 10 samples. In the digested solution, the concentrations of heavy metals, Pb, Cd, Cu, Zn, Cr, and Ni were determined by ICP-MS (Agilent 7500c, Oakland, CA, USA).

### 2.3. Evaluation of Heavy Metal Accumulation 

The geo-accumulation index (*I*_geo_) is commonly used to evaluate the accumulation level of heavy metals in soil and dust [26,27]. The calculation equation is as follows:

(1) $$I_{\mathrm{geo}}=log_{2}\left(\frac{C_{n}}{1.5B_{n}}\right)$$

where *C_n_* is the heavy metal content in soil or dust (mg·kg^−1^), and *B_n_* is the background content of heavy metal in the soil of Changsha (mg·kg^−1^), obtained from a reference [28]. For comparison, the soil background values were also used to assess the *I*_geo_ values of heavy metals in the dust. According to the *I*_geo_ value, the accumulation of heavy metals can be divided into different levels, that is, *I*_geo_ ≤ 0 indicates uncontaminated, 0 < *I*_geo_ ≤ 1 indicates uncontaminated to moderately contaminated, 1 < *I*_geo_ ≤ 2 indicates moderately contaminated, and 2 < *I*_geo_ ≤ 3 indicates moderately to heavily contaminated, 3 < *I*_geo_ ≤ 4 indicates heavily contaminated, 4 < *I*_geo_ ≤ 5 indicates heavily to extremely contaminated, and *I*_geo_ > 5 indicates extremely contaminated.

### 2.4. Probabilistic Risk Assessment Methods for Soil Heavy Metals

#### 2.4.1. Health Risk Assessment

The health risk model recommended by the United States Environmental Protection Agency (USEPA) has been widely used to evaluate the long-term exposure risk of heavy metals in urban soil and dust [29]. The model evaluates the risk by calculating the intake dose of heavy metals through three exposure methods: accidental oral ingestion, dermal contact, and inhalation [30]. Excessive intake of lead, cadmium, zinc, and copper in the body can cause a series of diseases, such as nerve and blood system damage, bone loss, liver damage and high blood pressure, but the carcinogenic toxicity of these heavy metals is still unclear [31]. Studies have shown that children generally face a higher risk from heavy metals than adults [32]. Therefore, we evaluated the noncarcinogenic health risks of heavy metals in urban soils and dust on children. The risk assessment models are as follows:

(2) $$HQ_{\mathrm{ingestion}}^{i}=\frac{C_{i}\times IR_{S}\times EF\times ED}{BW\times AT\times RfD_{\mathrm{ingestion}}}\times 10^{-6}$$

(3) $$HQ_{\mathrm{dermal}\;}^{i}=\frac{C_{i}\times ABS\times EF\times ED\times SA\times AF}{BW\times AT\times RfD_{\mathrm{dermal}\;}}\times 10^{-6}$$

(4) $$HQ_{\mathrm{inhalation}}^{i}=\frac{C_{i}\times IR_{a}\times EF\times ED}{BW\times AT\times PEF\times RfD_{\mathrm{inhalation}}}$$

(5) $$HQ\left(i\right)=HQ_{\mathrm{ingestion}}^{i}+HQ_{\mathrm{dermal}\;}^{i}+HQ_{\mathrm{inhalation}}^{i}$$

(6) $$\mathrm{HI}=\sum^{\;}HQ\left(i\right)$$

where, $HQ_{\mathrm{ingestion}}^{i}$, $HQ_{\mathrm{dermal}\;}^{i}$, $HQ_{\mathrm{inhalation}}^{i}$ represent the noncarcinogenic risk (unitless) of heavy metal *i* under the three exposure routes of accidental oral ingestion, dermal contact, and inhalation, respectively. HQ(*i*) represents the total health risk of heavy metal *i* under the three exposure pathways. *Ci* (mg·kg^−1^) represents the content of heavy metal *i* in soil or dust. *IR_S_* (mg·day^−1^) represents the intake rate through oral ingestion. *IR_a_* (m3·day^−1^) represents the inhalation rate. *EF* (day·year^−1^) represents the number of days of exposure per year, *ED* (year) represents the duration of exposure. *SA* (cm^-^·day^-^) represents the area of bare skin. *AF* (mg·cm^−2^) stands for skin adhesion factor. ABS (no unit) stands for skin absorption factor. This study used the default values for these parameters [33,34,35], which were 200 (mg·day^−1^) for *IRS*, 7.6 (m^3^·day^−1^) for *IR_a_*, 350 (day·year^−1^) for *EF*, 6 (year) for *ED*, 2800 (cm^2^·day^−1^) for *SA*, 0.2 (mg·cm^2^) for *AF*, and 0.001 (no unit) for *ABS*. *BW* represents the average weight, which is 15 kg for children. AT represents the average exposure time, and the default value is 6 × 365 (day). *RfD* (mg·kg^−1^ day^−1^) represents the reference dose of daily intake, and the value is shown in Table 1. HI represents the total health risk of heavy metals to children.

#### 2.4.2. Monte Carlo Simulation

Before the health risk assessment, we performed a Monte Carlo simulation to obtain the probability distribution of heavy metal concentrations in soil and street dust with different land uses. The Monte Carlo simulation adopted the statistical values of heavy metal concentrations, i.e., average value, minimum, maximum, and standard deviation [30]. The simulation was set to logarithmic distribution and ran 10,000 times for each element and land use. The Monte Carlo simulation was conducted by using Oracle Crystal Ball (Oracle Corporation, Redwood Shores, CA, USA).

### 2.5. Statistical Analysis

The correlation analysis was conducted using SPSS ver. 19.0 (IBM, New York, NY, USA). *I*_geo_ and HI values were calculated using Excel 2016 (Microsoft, Redmond, WA, USA). The sampling map was drawn using ArcGIS 10.2 (ESRI, Redlands, CA, USA). Other statistical graphs were drawn using Sigmaplot ver. 12.0 (Corel Corporation, Ottawa, AB, Canada) and Prism 9.0.0 (GraphPad, San Diego, CA, USA).

## 3. Results and Discussions

### 3.1. Characteristics of Heavy Metals in the Soil and Street Dust

The average contents of Pb, Cd, Cu, Zn, Cr, and Ni in soil samples from Changsha were 45.3, 0.69, 46.3, 220.4, 128.7, and 32.9 mg·kg^−1^, respectively, which were 1.61, 5.75, 1.78, 1.78, 2.98, 1.37 and 1.22 times the corresponding background values, respectively (Table 2). The contents of heavy metals varied largely in the soils. The variation coefficients of Cd, Pb, Zn and Cr were 58.2%, 44.6%, 41.6% and 40.3%, respectively. The soil samples were collected from urban green spaces and suburban farmland. The large variations of these metals were most likely caused by human activities. The accumulation of heavy metals in the soils was attributed to the development of non-ferrous smelting and chemical industries (i.e., chromium salt plants) in Changsha. The dense traffic in the urban area causes Cd, Pb, Cu and Zn emissions to the environment, eventually accumulating in the soil.

The average contents of Pb, Cd, Cu, Zn, Cr, and Ni in the dust samples in Changsha were 130.1, 3.9, 130.8, 667.2, 223.2, and 50.5 mg·kg^−1^, respectively, which were significantly higher ratios than that in the soil samples (*p* < 0.05). The street dust in the urban area may have come from industrial and traffic emissions, re-suspension of soil and weathered materials, etc. [36,37]. Studies have shown that street dust with a smaller particle size contains higher levels of heavy metals and is easier to migrate over longer distances [8]. After entering the soil, the dust would be adsorbed by soil organic matter and clay minerals, and the heavy metals would be diluted in the soil. Hence the heavy metal contents of street dust are usually higher than those in soil [19]. The variation coefficients of heavy metals in street dust samples ranged from 72.1 to 141.8%, much higher than those in urban soils. Rainfall, strong wind, and cleaning activities will refresh the street dust on the road [23]. The heavy metal contents of street dust vary spatially and change with time. It was suggested that the heavy metal contents in street dust are more susceptible to natural and human disturbances [38].

The heavy metal contents in the soil and dust samples are comparable to those reported in the other major cities, such as Beijing [39,40], Shanghai [41,42] and Nanjing [43,44]. However, the heavy metal contents reported in cities with heavy industry are often higher than those in Changsha. For example, the average contents of Pb (466.6 mg·kg^−1^) and Zn (564 mg·kg^−1^) in the soil of Xiangtan city, and Cu (111.3 mg·kg^−1^) and Cd (1.32 mg·kg^−1^) in the soil of Kunming city were significantly higher than those in the soil of Changsha [45,46]. The average content of Pb (956 mg·kg^−1^), Zn (2379 mg·kg^−1^) and Cd (139 mg·kg^−1^) in street dust of Zhuzhou city, and the Zn (1019.8 mg·kg^−1^), Cr (401.6 mg·kg^−1^), and Cu (240.9 mg·kg^−1^) in street dust of Luoyang city were significantly higher than those in the street dust of Changsha [22,47]. On the contrary, heavy metal contents in soil and street dust of cities with less industrial production, such as Zahedan (Iran) [20], Lublin (E Poland) [38], Malayer (Iran) [48] and Novi Sad (Serbia), were generally lower than those in Changsha [49]. It was suggested that the urban industry is a primary factor affecting the contents of heavy metals in urban soil and street dust [8].

### 3.2. Impacts of Land Uses on Heavy Metals

Significant differences were found in the heavy metal contents between land uses in Changsha (*p* < 0.05). The contents of Pb, Cd, Cu, Zn, Cr, and Ni in the park and roadside soils were generally higher than that in residential and suburban agricultural areas (Table 3). The *I*_geo_ values of Pb, Cd, Cu, Zn, and Cr in the soil of parks and roadsides exceeded 0, indicating that these are accumulated to varying degrees (Table 4). The *I*_geo_ values of Cd in the four land uses and Zn in the park soil were greater than 1, indicating moderate to heavy contamination. Compared with other land uses, the urban parks sampled in this study have long histories with little soil disturbance. Heavy metals have been continually accumulating in the park soil for a long time. The weathering of paint and coatings on the surface of old buildings in the parks may be an additional source of heavy metals. Wang [15] showed that the contents of heavy metals in parks of Beijing were significantly higher than in other areas. Lu [50] found that the old urban area of Nanjing had higher heavy metal contents than the new urban area. In the past, there were many non-ferrous metal smelting companies and chromium salt plants in Changsha, but most of them have been closed due to environmental policies. The industrial production and transportation of industrial raw materials may produce emissions, causing heavy metals to accumulate in the soil [51]. Because the old residential areas have tiny green spaces, the residential areas sampled in this study have a relatively shorter built history than the parks, resulting in low heavy metal contents in the soil. In short, the contents of heavy metals in soils with different land uses were affected by the age of sampled green spaces and the intensity of the emission sources.

Compared with the soil, variations of the heavy metal contents in street dust between land uses become larger (Table 3). The contents of heavy metals in the street dust of urban parks, roadsides, and residential areas were higher than those of street dust in suburban agricultural areas. There was no significant difference in the content of heavy metals in street dust between urban parks, roads and residential areas (*p* > 0.05). This was attributed to the large variability of the heavy metal contents, such that the variation coefficient of Zn in the street dust of the parks was higher than 100%. Many sources of heavy metals in street dust can be found in an urban area, including as smelting fumes, wear of motor vehicles and road materials, weathering of building coatings and paint. Due to the industrial activities and transportation in cities, the heavy metal contents of street dust in urban areas was generally higher than that in suburban agricultural areas. The *I*_geo_ values of Cd in street dust in the four land uses were between 3 to 5, and the *I*_geo_ values of the Zn in parks, roads and residential areas and of the Pb in residential areas were between 2–3. The *I*_geo_ values of Cu in street dust were between 1–2 (Table 4). It is suggested that the street dust in urban areas was heavily contaminated with Cd, Zn, Pb and Cu.

Some sites had high levels of heavy metals in street dust, but the soil at the same sites does contain low levels of heavy metals, which may be related to recent industrial production activities or transportation in their surroundings. For example, the sampling sites with the highest Pb, Cd, Cu, and Zn content in street dust had 465.6, 13.55, 489.9, and 4497.2 mg·kg^−1^, respectively, and the corresponding Pb, Cd, Cu, and Zn content in the soil at the same sampling sites were only 45.9, 0.95. 40.7 and 199.4 mg·kg^−1^. Street dust is easily disturbed by climatic conditions and human activities, and recent industrial emissions and transportation activities significantly impact the content of heavy metals in street dust. Short-term pollution caused the contents of heavy metals in street dust to be very different from nearby soil. The dilution effect of soil parent material and organic matter can also be a cause for the low contents of heavy metals in the soil samples.

### 3.3. Correlations of Heavy Metals in Soil and Street Dust

**Figure 2:**
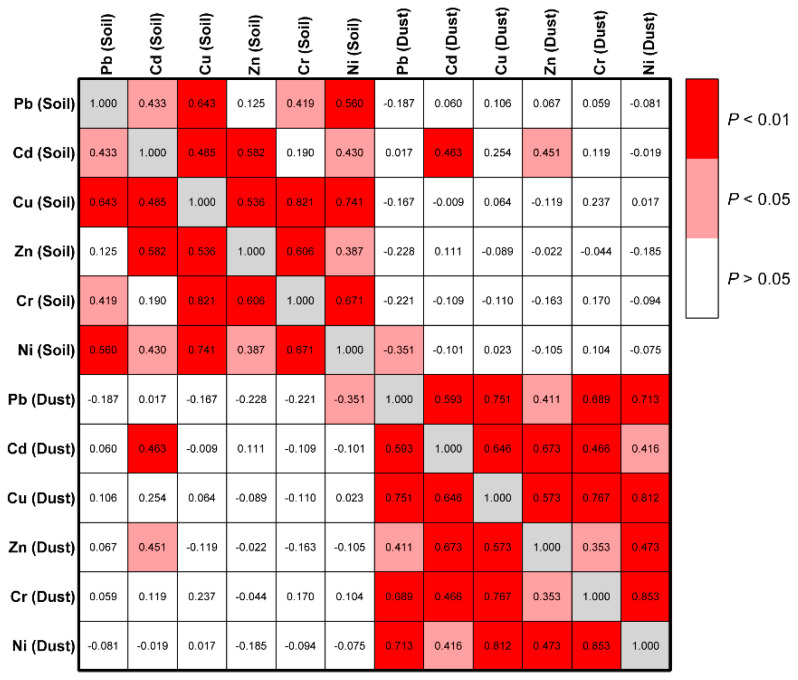
Correlations of heavy metals in the soil and street dust of Changsha.

Significant correlations can be found between all heavy metals in the street dust (Figure 2). Except for Cd and Cr, Pb and Zn, significant correlations can also be found between the heavy metals in the soil. The results suggested that these heavy metals have similar sources in the city, such as emissions from industrial plants and traffic. The heavy metals in urban soil and street dust mainly come from atmospheric particulate deposition, and particulates from different sources will be mixed during atmospheric transportation, causing the contents of heavy metals to correlate [52]. Cr in the soils mainly comes from industrial sources (e.g., chromium salt plants), Pb may come from early Pb-containing gasoline and paint erosion, while Cd and Zn mainly emit from smelters and transportation [53,54]. Leaded gasoline has been banned in China since 2000. After that, less Pb would be released from traffic. The difference in the sources may be the cause of the weak correlations between Cd and Cr, Pb and Zn in the soil.

**Figure 3:**
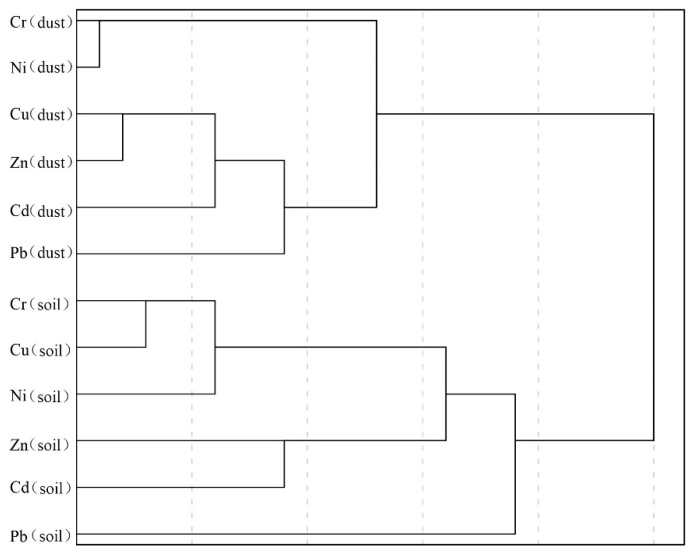
Cluster analysis of heavy metals in the soil and street dust of Changsha.

The contents of heavy metals in the street dust were uncorrelated with those in the soils, except for the correlation of Cd in soil with Cd and Zn in dust (Figure 2). Similarly, the cluster analysis divided the heavy metals in the street dust and soil into two categories (Figure 3). The results indicated that the distribution of heavy metals in the street dust is quite different from that in the soils, e.g., some sites have high heavy metal contents in street dust but low contents in the soil. The heavy metal contents in street dust are dominated by the recent emissions, easily changed with time and disturbance. On the contrary, heavy metals in the soil result from long-term accumulation, reflecting the intensity of long-term pollution emissions. The contents of heavy metals in the soil were also affected by soil parent materials. Although street dust deposition is one of the main inputs of heavy metals in soil, the content of heavy metals in street dust does not necessarily correlate to that in the soil.

### 3.4. Health Risk Assessment of Heavy Metals in Soil and Street Dust

**Figure 4:**
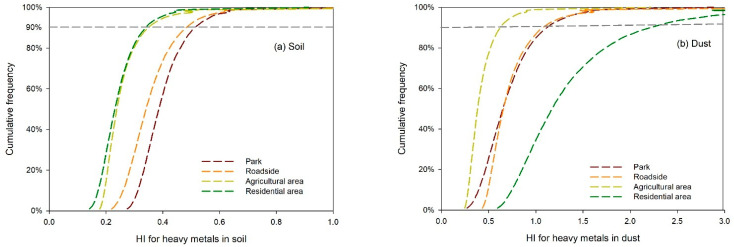
Cumulative frequency of noncarcinogenic risk of heavy metals in soil (**a**) and street dust (**b**).

The health risk assessment showed that the 90th percentile HI value of heavy metals in the soils was 0.448 (Table 5), lower than the safety threshold of 1, which indicates the health risk was at a safe level. The HI values of heavy metals in soil varied with land uses following the order of roads > parks > agricultural area and residential areas (Figure 4a). The 90th percentile HI values were less than 1 in all the land uses, suggesting a low risk posed by heavy metals in the soils. The 90th percentile HI value of heavy metals in the street dust was 1.449 (Table 5), higher than 1, indicating potential health risks. The HI values were ranked residential area > roads and parks > suburban agricultural areas (Figure 4b). The results suggested that the street dust in residential areas poses the highest health risks to children. The sampled residential areas are often full of parked vehicles. The parking process will increase the wear of tires and brake pads, resulting in a large amount of heavy metals being released [55]. In the current study, a Monte Carlo simulation was adopted to evaluate the probability distribution of the health risks. However, the limited number of samples may not reflect the spatial and temporal changes in health risks posed by heavy metals in the soil and dust. Time-series sampling may be helpful for future research. In all, attention should be paid to the pollution and health effects of street dust in urban residential areas.

Pb and Cr in the soils contributed 59% and 30% to the total risk, respectively, while Pb, Cr and Cd in the street dust contributed 62%, 20%, and 8% to the total risk, respectively (Table 5). It is suggested that Pb, Cr and Cd are the main risk elements in Changsha. The primary sources of Pb, Cr and Cd are the chromium salt plants and non-ferrous smelting plants. It is necessary to strengthen the emission supervision of these industrial enterprises. In terms of exposure route, accidental ingestion contributed the highest health risk. We assessed the health risks to children, who are more likely to be exposed to soil and street dust through hand-to-mouth behavior, resulting in high oral exposure doses. Reducing the time children play on the ground and washing their hands in time would reduce the noncarcinogenic risk posed by heavy metals in the soil and street dust.

## 4. Conclusions

The heavy metal contents in soil and street dust with different land uses were investigated in Changsha. The average contents of Pb, Cd, Cu, Zn, Cr and Ni in the soils were 45.3, 0.69, 46.3, 220.4, 128.7 and 32.9 mg·kg^−1^, respectively, higher than their corresponding background values but considerably lower than the values in the street dust, which were 130.1, 3.9, 130.8, 667.2, 223.2, 50.5 mg·kg^−1^, respectively. The soils in the parks and roadsides had higher heavy metal contents than those in residential and agricultural areas, mainly attributed to the long history of parks and the high emissions around the roads. The street dust collected from parks, roadsides and residential areas contained higher heavy metal contents than agricultural areas. Street dust is easily affected by road cleanliness, rainfall, strong winds and other disturbances, which mainly reflect pollution emissions in a short period. Most of the heavy metals in the soil were uncorrelated with those in the street dust. The health risks posed by heavy metals in the soil were acceptable, but the risk posed by street dust threatens the health of children, especially the street dust in residential areas. Pb, Cr and Cd are the main risk contributors. Reducing the frequency of children playing on the ground and emissions from industrial plants and traffic would reduce the risk of exposure to heavy metals in soil and street dust.

## Figures and Tables

**Table 1:** Summary of daily reference dosage for heavy metals (mg·kg^−1^ day^−1^) ^a^.

	Pb	Cd	Zn	Cu	Cr (VI)	Ni
*RfD_ing_*	3.5 × 10^−3^	1.0 × 10^−3^	3.0 × 10^−1^	4.0 × 10^−2^	3.0 × 10^−3^	2.0 × 10^−2^
*RfD_dermal_*	5.3 × 10^−4^	1.0 × 10^−5^	6.0 × 10^−2^	1.2 × 10^−2^	6.0 × 10^−5^	5.4 × 10^−3^
*RfD_inhal_*	2.0 × 10^−4^	1.0 × 10^−5^	6.0 × 10^−2^	4.2 × 10^−2^	2.9 × 10^−5^	1.4 × 10^−5^

^a^ Daily reference dosages for Cu, Mn, Ni, Cd, Pb, and Zn are cited from reference [33], and Cr and As are cited from reference [32]; the content of Cr (VI) is assumed to be 1/7 of the total Cr content [29].

**Table 2:** Statistical results of heavy metal content in soil and street dust in Changsha.

	Soil (mg·kg^−1^)						Street Dust (mg·kg^−1^)					
	Pb	Cd	Cu	Zn	Cr	Ni	Pb	Cd	Cu	Zn	Cr	Ni
Average	45.3	0.69	46.3	220.4	128.7	32.9	130.1	3.91	130.8	667.2	223.2	50.5
Median	44.6	0.61	43.8	213.5	122.8	33.9	101	2.59	106.8	446.5	176.9	32.6
Standard deviation	20.2	0.38	13.8	91.6	51.9	9.1	102.1	3.08	94.3	804.8	178.1	69.6
Minimum	11.3	0.14	15.3	102.4	33.5	9.8	6.5	1.00	15.0	97.9	19.9	14
Maximum	90.6	1.55	72.4	482.6	258.6	52.3	465.6	13.55	489.9	4497.2	1005	405.8
Variation coefficient (%)	44.6	58.2	29.8	41.6	40.3	27.7	78.5	78.8	72.1	120.6	79.8	137.8
Kurtosis	−0.65	−0.37	−0.28	1.31	0.07	0.12	3.57	2.19	5.49	16.94	11.69	24.3
Skewness	0.32	0.65	0.08	1.13	0.57	−0.18	1.86	1.56	1.81	3.7	2.97	4.7
Background values ^a^	28	0.12	26	74	94	27						

^a^ Background values are cited from the reference [28].

**Table 3:** Concentrations of heavy metals in the soil and street dust with different land uses of Changsha.

	Pb	Cd	Cu	Zn	Cr	Ni
Soil (average ± standard deviation, mg·kg^−1^)						
Park	60.2 ± 18.9 a	1.05 ± 0.29 a	52.8 ± 10.9 ab	287.2 ± 102.5 a	153.3 ± 45.3 ab	37.1 ± 5.0 a
Roadside	47.5 ± 18.6 ab	0.70 ± 0.38 b	54.2 ± 12.9 a	235.4 ± 101.1 ab	163.0 ± 61.1 a	39.1 ± 5.4 a
Agricultural area	36.2 ± 20.3 b	0.47 ± 0.29 b	40.8 ± 12.3 b	214.4 ± 56.9 ab	111.8 ± 28.8 bc	26.5 ± 3.8 b
Residential area	37.4 ± 20.2 b	0.52 ± 0.26 b	37.3 ± 12.5 b	144.4 ± 34.8 b	86.5 ± 30.2 c	28.7 ± 12.9 ab
Street dust (average ± standard deviation, mg·kg^−1^)						
Park	101.8 ± 51.6 a	6.38 ± 4.22 a	129.8 ± 88.2 ab	763.0 ± 426.8 a	195.3 ± 113.0 a	33.8 ± 14.5 a
Roadside	101.0 ± 48.7 a	2.64 ± 1.41 a	148.8 ± 51.4 a	982.2 ± 1423.9 a	282.6 ± 133.5 a	47.3 ± 24.4 a
Agricultural area	73.5 ± 73.4 b	2.10 ± 1.22 a	58.2 ± 21.5 b	188.7 ± 171.0 a	112.3 ± 36.1 b	20.9 ± 5.9 a
Residential area	244.1 ± 139.0 a	4.52 ± 2.80 a	186.3 ± 136.3 ab	734.8 ± 488.9 a	302.6 ± 287.1 a	98.1 ± 126.8 a

Note: The same letter means no significant difference, and different letters indicate significant difference, *p* < 0.05.

**Table 4:** Average *I_geo_* values of heavy metals in the soil and street dust with different land uses of Changsha.

	Soil (mg·kg^−1^)						Street Dust (mg·kg^−1^)					
	Pb	Cd	Cu	Zn	Cr	Ni	Pb	Cd	Cu	Zn	Cr	Ni
Park	0.44	2.50	0.41	1.30	0.06	−0.14	0.87	4.77	1.25	2.53	0.11	−0.38
Roadside	0.04	1.77	0.44	0.98	0.11	−0.06	1.13	3.73	1.86	2.49	0.86	0.11
Residential area	−0.32	1.36	−0.16	0.34	−0.81	−0.63	2.30	4.40	1.96	2.35	0.78	0.64
Agricultural area	−0.38	1.17	0.02	0.89	−0.38	−0.62	0.65	3.39	0.48	0.47	−0.40	−1.00

**Table 5:** The 90th percentile HQ and HI values for heavy metals in the soil and street dust of Changsha.

	HQ_Pb_	HQ_Cd_	HQ_Cu_	HQ_Zn_	HQ_Cr_	HQ_Ni_	RfD_ing_	RfD_dermal_	RfD_inhal_	HI
Soil	0.264	0.019	0.018	0.014	0.135	0.026	0.423	0.024	0.001	0.448
Street dust	0.903	0.115	0.076	0.059	0.287	0.063	1.368	0.075	0.004	1.449

## Data Availability

The data presented in this study are available on request from the corresponding author.
